# A Brief Review of Mental Health Issues among Asian and Pacific Islander Communities in the U.S.

**DOI:** 10.31372/20200504.1124

**Published:** 2021

**Authors:** Mijung Park

**Affiliations:** University of California, San Francisco, United States

**Keywords:** Asian, Pacific Islander, mental health issue, mental health disparities, mental health services disparities

## Abstract

The purpose of this paper is to provide a brief summary of mental health issues among Asian and Pacific Islander (API) communities in the U.S. APIs include individuals from Far East Asia (e.g., Korea, China), Central Asia (e.g., Afghanistan, Uzbekistan), South Asia (e.g., India, Pakistan), South East Asia (e.g., Thailand, Philippines), Western Asia (e.g., Iran, Saudi Arabia), and Pacific islands (e.g., Hawaii, Samoa, Mariana island, Fiji, Palau, French Polynesia, Marshall Islands, Micronesia, New Zealand, Tokelau islands, Niue, and Cook Islands). Collectively they speak more than one hundred languages and dialects. Such a diversity across the API community presents unique challenges and opportunities for research, education, and practice. The existing body of literature on mental health issues in API communities is marred by the lack of high-quality data and insufficient degrees of disaggregation. Such a knowledge gap hindered our ability to develop culturally and linguistically tailored interventions, and in turn, API communities have experienced mental health disparities and mental health services’ disparities. To move the field forward, future research effort with APIs should focus on articulating variations across different API subgroups, identifying what explains such variations, and examining the implications of such variations to research, practice, education, and policy.

Asian and Pacific Islander (API) communities include a wide range of historical, cultural, and ethnic heritages. API includes individuals from Far East Asia (e.g., Korea, China), Central Asia (e.g., Afghanistan, Uzbekistan), South Asia (e.g., India, Pakistan), South East Asia (e.g., Thailand, Philippines), Western Asia (e.g., Iran, Saudi Arabia), and Pacific islands (e.g., Hawaii, Samoa, Mariana island, Fiji, Palau, French Polynesia, Marshall Islands, Micronesia, New Zealand, Tokelau islands, Niue, and Cook Islands). The diversity across the API communities presents unique challenges and opportunities for research, education, and practice. The purpose of this paper is to evaluate what we know about mental health issues among the API communities.

## Demographic Characteristics of the API Communities in the United States

In the U.S., Asian Americans (AAs) represent the fastest growing minority group. Between 2000 and 2015, the number of AAs grew from 11.9 million to 20.4 million. By 2065, Asians are projected to become the largest immigrant group in the nation, making up 38% of all immigrants in the U.S. (López, Ruiz, & Patten, 2017). AAs include forty-three heterogeneous communities with distinctive language, religion, and socio-cultural heritages. They speak more than one hundred languages and dialects. While no single country-of-origin dominates, the largest AA subgroups are people of Chinese, Indian, Filipino, Vietnamese, and Korean origin. Furthermore, immigration history varies among AA subgroups. For example, while less than 30% of Japanese Americans are immigrants, more than 90% Bhutanese are foreign born.

AAs have been portrayed as a population with high education and middle-class earning potential. The *model minority* myth refers to the misconception that all AAs are well adjusted and thriving. Such notions overlook the heterogeneity within the AA community. As a consequence, AAs are often left out of national conversation on poverty and on mental health needs. In fact, poverty rates vary significantly among different AA subgroups. For example, in 2017, about 10% AAs experienced poverty. During the same year, about 6.0% of Filipinos experienced poverty, compared to 16.2% of Hmongs and 15% of Koreans (United States Census Bureau, 2018). Poverty, social isolation, limited English proficiency, and inadequate community outreach keep particularly vulnerable AA subgroups from accessing social and mental health services. Strong family support is often cited as the major strengths among AAs. However, not all AA individuals enjoy a robust social support.

Pacific Islanders (PIs) make up about 0.5% of the U.S. population. Between 2000 and 2010, PIs living in the U.S. have grown 40% (Office of Minority Health, 2020). Similar to AAs, PIs comprise diverse ethnic and cultural subgroups. Each group enjoys unique cultural, tradition, and historical circumstances. In 2017, 15.4% of Native Hawaiians/Pacific Islanders, in comparison to 9.6% of non-Hispanic whites, were living at the poverty level. In the same year, the unemployment rate for Native Hawaiians/Pacific Islanders was 5.8%, as compared to 4.2% for non-Hispanic whites (Fontenot, Semega, & Kollar, 2018).

## Psychiatric Morbidities in API Communities

Nationally, representative epidemiological data for mental health issues among APIs are sparse. Furthermore, when data on APIs are collected, it is often not broken down for subgroups. Moreover, the available disaggregated data are mainly focused on the largest subgroups (e.g., Chinese and Filipinos), making it difficult to capture meaningful information about subgroups with smaller population sizes.

Existing national study suggests that APIs experience a similar or a lower rate of mental health issues compared to their non-Hispanic White counterparts (Office of Minority Health, 2020). According to a large epidemiological study, about 17.30% of AAs met DSM diagnostic criteria for at least one psychiatric disorder in their life time, and about one in ten AAs could be diagnosed with at least one psychiatric disorder over the past 12 months (Spencer, Chen, Gee, Fabian, & Takeuchi, 2010). In 2018, about 2.1% of PIs—compared to 3.7% of non-Hispanic White—reported serious psychological distress in the past 30 days. About 6.9% of PI adults—compared to 7.8% of non-Hispanic White adults—had major depressive episode (Office of Minority Health, 2020).

Aggregated data presented in the previous section, however, do not show the variations in psychiatric morbidities across various API subgroups. For example, in California, Filipino and Korean older adults were more likely to report symptoms indicative of mental health issues than their non-Hispanic white counterparts (Sorkin, Nguyen, & Ngo-Metzger, 2011). In another study, older Vietnamese Americans experience higher rates of major depression compared to their Chinese and Filipino counterparts (Gonzalez, Tarraf, Whitfield, & Vega, 2010). In a different study, about 13.7% of Native Hawaiian adults living in Hawaii met the diagnostic criteria for depressive disorders (Hawaii Health Data Warehouse; Hawaii State Department of Health, 2016).

The prevalence of psychiatric morbidities among API communities may be severely underestimated due to the lack of proper diagnostic tools for this culturally and linguistically diverse population. Additionally, individuals from different culture may experience and exhibit symptoms of psychiatric conditions differently. Therefore, diagnostic criteria developed predominantly with European Americans may not capture the variations of psychiatric symptoms across different cultural subgroups. Moreover, attitudes towards mental health issues and stigma may lead to underreporting psychiatric symptoms in these populations.

## Mental Health Services Disparities among API Communities

In addition to differential mental health prevalence, mental health services disparities among APIs of all age groups have been extensively documented (Byers, Lai, Nelson, & Yaffe, 2017; Choi & Gonzalez, 2005; Cook, Trinh, Li, Hou, & Progovac, 2017; Mackenzie, Pagura, & Sareen, 2010; Marshall et al., 2006; Shin, 2002). AAs are between two and five times less likely to receive mental health services than their non-Hispanic White counterparts. In 2018, about 10.9% of PIs received mental health services in the past year, compared to 18.6% of non-Hispanic White. About 6.3% of PIs received prescription medications for mental health treatment or counseling, compared to 15.4% of non-Hispanic White.

Scholars have proposed several reasons for mental health services disparities that API communities experience, including the lack of culturally and linguistically appropriate mental health care services available for this population, the lack of insurance coverage, limited economic resources, stigma around mental health issues, culturally unique help seeking behaviors, and differential expectation about mental health care services (Cook et al., 2017; Takeuchi et al., 2007).

## Conclusion

In conclusion, the existing body of literature on mental health issues among API communities is marred by the lack of high-quality, up-to-date data and insufficient degrees of disaggregation. Particularly, PI communities suffer severely from the lack of epidemiological data as well as from research on intervention tailored for this community. Such a knowledge gap hinders our ability to develop culturally and linguistically tailored intervention, and in turn, API communities has been subject to mental health disparities and mental health services disparities. To move the field forward, future research effort with APIs should include articulating variations across different API subgroups, identifying what explains such variations, and examining the implications of such variations to research, practice, education, and policy.

## Declaration of Conflicting Interests

The author declares no conflicts of interest with respect to the work, authorship, and/or publication of this article.
